# 3D connective micro-fragment enriched with stromal vascular fraction in osteoarthritis: chondroprotective evidence in a preclinical *in vivo* model

**DOI:** 10.3389/fcell.2025.1533405

**Published:** 2025-02-27

**Authors:** Giovanna Desando, Matilde Tschon, Lucia Martini, Maria Sartori, Gianluca Giavaresi, Milena Fini, Antonella Cellamare, Carlo Soranzo, Cristina Longinotti, Martina D’Alessandro, Livia Roseti, Brunella Grigolo

**Affiliations:** ^1^ IRCCS Istituto Ortopedico Rizzoli, Laboratorio RAMSES, Bologna, Italy; ^2^ IRCCS Istituto Ortopedico Rizzoli, Scienze e Tecnologie Chirurgiche, Bologna, Italy; ^3^ IRCCS Istituto Ortopedico Rizzoli, Scientific Director, Bologna, Italy; ^4^ Fidia Farmaceutici S.p.A., Padova, Italy

**Keywords:** adipose stromal cells, articular cartilage repair, cell-based therapies, preclinical *in vivo* study, osteoarthritis, stromal vascular fraction, connective tissue micrograft

## Abstract

**Introduction:**

Adipose-derived cell therapies are one of the most common regenerative therapeutic options to alleviate the multi-component damage of osteoarthritis (OA). Adipose stromal vascular fraction (SVF) has gained scientific consensus for its ability to interact protectively with the joint microenvironment. Recently, the wide range of enzyme-free tissue processing systems has outperformed classical treatments, because of their ability to produce connective micrografts enriched with the SVF (mctSVF). This preclinical *in vivo* study evaluates the chondroprotective potential of a newly generated mctSVF compared with *in vitro* expanded adipose stromal cells (ASC) in OA.

**Methods:**

A mild grade of OA was induced through bilateral anterior cruciate ligament transection (ACLT) surgery in 32 Specific Pathogen Free (SPF) Crl: KBL (NZW) male rabbits followed by the surgical excision of inguinal adipose tissue. After 2 months, OA joints were treated with an intra-articular (IA) injection of mctSVF or ASC. Local biodistribution analysis was used to determine migration patterns of PKH26-labelled cells in the knee joint after 1 month. Efficacy was assessed by gross analysis, histology and immunohistochemistry on the osteochondral unit, synovial membrane and meniscus.

**Results:**

We elucidate the effectiveness of a one-step approach based on mechanical isolation of mctSVF. Through epifluorescence analysis, we found a similar pattern of cell distribution between cell treatments, mainly towards articular cartilage. Similar regenerative responses were observed in all experimental groups. These effects included: (i) osteochondral repair (promotion of typical anabolic markers and reduction of catabolic ones); (ii) reduction of synovial reactions (reduced synovial hypertrophy and inflammation, and change of macrophage phenotype to a more regenerative one); and (iii) reduction of degenerative changes in the meniscus (reduction of tears).

**Discussion:**

Our study demonstrates the validity of a novel mechanical system for the generation of the mctSVF micrograft with chondroprotective potential in a preclinical model of moderate OA. The resulting final product can counteract inflammatory processes beyond the OA microenvironment and protect cartilage through the colonization of its structure. The intact and active microanatomy of mctSVF makes it a suitable candidate for translational medicine to treat OA without the need for cell manipulation as with ASC.

## 1 Introduction

The multicomponent nature of osteoarthritis (OA) pathogenesis poses a major orthopaedic challenge because of the limited benefit of current clinical interventions (Vrouwe et al., 2021). In this context, cell-based therapeutics have attracted great attention for their pleiotropic wound-healing effects not only on cartilage but also on various joint tissues, affected during OA (Djouad et al., 2009; Filardo et al., 2013; Vinatier and Guicheux, 2016). While clinical studies on bone marrow and adipose tissue, the most common tissue sources for regenerative medical purposes (Zhang and Schon, 2022), have shown promising chondroprotective properties, it is still debated which of them will best counteract the hallmarks of OA (Beane et al., 2014; El-Badawy et al., 2016; Gun-II Im et al., 2005). However, the minimally invasive nature of adipose tissue harvesting, the wide range of isolation methods (Cicione et al., 2023; Gentile et al., 2019; Ossendorff et al., 2023), and the highest yield of mesenchymal stromal cells have led to its more prevalent use than bone marrow (Ibrahim et al., 2022; Oberbauer et al., 2015).

The classical enzymatic procedure, which includes treatment of the tissue with clinical-grade collagenases and/or dispases, permits the SVF first and then adipose stromal cells (ASC) isolation and subsequent culture expansion. Good clinical results have been reported using expanded ASC to improve joint pain and function (Perucca Orfei et al., 2023; Perdisa et al., 2015). Despite their benefits, the long-term cell culture and the need for two-step procedures make them time-consuming, increasing the risk of potential cell changes in apoptosis and senescence (Danisovic, 2017; Froelich et al., 2013) and the need for a GMP cleanroom (Si et al., 2019). On the other hand, the techniques available for mechanically disaggregating adipose tissue into fragments require various steps (centrifugation, filtration, and washing) (Greenwood et al., 2022; Aronowitz JA, 2014), by overcoming some of the limitations associated with the use of expanded cells. Typically, the final products from mechanical isolation may encompass clusters of free cells or fragmented adipose tissue, characterized by a structural connective tissue with its ASC-rich derivatives (mainly SVF) (Francesco et al., 2021). With a size range from micrometres to nanometers, which depends on the filtration system chosen, these components present a significant advantage: they are effectively retained in the OA knee joint, enhancing their therapeutic potential (Cicione et al., 2023; Gentile et al., 2019; Ossendorff et al., 2023). Of note, the various mechanical approaches available may be more advantageous than enzymatic isolation because they avoid the use of exogenous enzymes and preserve the native three-dimensional (3D) structure with intrinsic cues. However, they pose the need for rigorous preclinical studies to select the most promising clinical solutions (Francesco et al., 2021; Busato et al., 2021; Trivisonno et al., 2019; Aronowitz et al., 2015; Aronowitz et al., 2015). In this direction, several studies have compared enzymatic and mechanical isolation techniques using different systems (such as Cytori, Celution, Lipogems, etc.) to better understand their molecular signatures for clinical applications. While mechanical isolation techniques typically yield fewer nucleated cells than enzymatic methods, they are generally safe, cost-effective, and quick to process (Aronowitz et al., 2015). Despite common features, there are differences between filtration systems in terms of: (i) fragment size; (ii) cell population maintained; (iii) paracrine properties; (iv) differentiation potential; and (v) immunomodulatory properties (Gentile et al., 2017; Uguten et al., 2024). Our previous *in vitro* research demonstrated similar outcomes between mechanical and enzymatic isolation methods, showing that both systems contain functional cell populations capable of supporting osteochondral regeneration (Desando et al., 2021). Given the promising *in vitro* evidence, our focus was to validate *in vivo* the characteristics of the Hy-Tissue SVF system (Fidia Farmaceutici, Abano Terme), a CE-marked class IIa device for microfractionation of adipose tissue, and its potential to treat mild OA. This interest stems from the potential benefits that may arise from the use of mctSVF to overcome some of the ethical, regulatory and safety concerns that may be associated with expanded ASC (Desando et al., 2021). Herein, we aimed to test the therapeutic potential of connective tissue micro-fragments enriched with SVF (mctSVF), as an optional treatment for OA, in a validated rabbit model allowing good prediction of tissue repair and clinical translatability. It was hypothesized that the mctSVF encased in native connective tissue would stabilize at the articular site of the knee and have a greater ability to heal than ASC treatment. By characterizing the response to intra-articular (IA) administration of mctSVF, we can conclude that this approach is effective in restoring the osteochondral unit and reducing inflammation, with the potential for clinical translation in this clinical setting.

## 2 Materials and methods

### 2.1 Ethics committee approval and experimental design

All *in vivo* procedures were performed following the animal care and use guidelines in compliance with the Italian Law (Law by Decree no 26/2014) and according to the European Commission Recommendation on guidelines for the accommodation and care of animals used for experimental and other scientific purposes (2007/526/EC). The *in vivo* protocol was developed following the PREPARE guidelines (Planning Research and Experimental Procedures on Animals: Recommendations for Excellence); it was approved by the Animal Welfare Body of IRCCS-Istituto Ortopedico Rizzoli (IOR) on 14th May 2019, authorized by the Italian Ministry of Health (Aut. n. 557/2019-PR on 23rd July 2019) and foresaw the efficacy testing of one-step and *in vitro* expanded cell-based and viscosupplementant products. To further improve the quality of this study, the *in vivo* research methodology has been reported according to criteria set in the Animal in Research: Reporting of *in Vivo* Experiments (ARRIVE 2.0) guidelines (Percie du Sert et al., 2020). The ARRIVE Essential checklist is provided in Supplementary Table 1. The sample size was calculated using the software G*Power v.3.1.9.2 (Franz Faul, Universität Kiel, Germany): by assuming that an effect f ≥ 0.63 could be obtained for Laverty’s histomorphometric score, in a one-way ANOVA model (type of treatment) with α = 0.05 and 1-β = 0.80, it was calculated that a minimum number of n = 8 animals per experimental group was required. Following the principle of the 3Rs, the sample size of the control groups, SHAM, and OA was reduced to n = 4 per group, considering previous studies (Desando et al., 2013; Grigolo et al., 2009) and literature efficacy studies (Mainil-Varlet et al., 2013) with the same animal model.

The present work reports data and results to compare ASC and mctSVF products in the treatment of rabbit knee OA versus the control groups. Animals received from the vendor with the same age were divided into 6 groups (OA (n = 4 + n = 9 from previous studies), SHAM (n = 4 + n = 7 from previous studies), ASC (n = 8), mctSVF (n = 8), PKH-26 ASC (n = 4), PKH26 mctSVF (n = 4)) and received the same stabling conditions. No randomization was applied to allocate animals to groups; however, outcome assessors (GD, AC) were blind to the allocation groups during the assessment and analysis of the data to minimize performance and detection biases. Animals in the same group receiving the same treatment were placed in closely spaced cages to minimise potential co-founders. No animal or data points were excluded before the final analysis. The primary outcome measure of this study was femoral and synovial histological analysis, supported by semi-quantitative scoring systems, to assess the efficacy of the 2 cell treatments.

### 2.2 Rabbit *in vivo* model and procedures

#### 2.2.1 Surgical OA induction

Thirty-two SPF Crl: KBL (NZW) male rabbits (Charles River Laboratories, Italy), 2.7 ± 0.2 kg b. w., were used and housed in single cages in standard controlled conditions with free access to water and pellet diet and specific enrichments. A 10-day conditioning period with frequent animal handling was pursued to acclimate animals.

Surgical procedures were aseptically performed after a 10-day quarantine period and under general anaesthesia induced by premedication with i. m. injections of ketamine (44 mg/kg) and xylazine (0.3 mg/kg) and maintained with O_2_/air and 2%–3% halogenate (isofluorane) in spontaneous breathing by a facial mask. A 2 cm surgical incision was bilaterally carried out in the lateral surface of the knee: after the articular capsule incision, the patellar bone was displayed medially, and the anterior cruciate ligament (ACL) was exposed and transected with the knee placed in full flexion. To avoid spontaneous healing, a small fragment of 2.5-mm length was removed. The articular capsule and skin were sutured by layers. In the SHAM group, skin and articular capsule incisions were performed, without ACL transection.

In the immediate post-op, i. m. injection of anaesthetics (3.75 mg of a solution 7.5 mg/mL of ropivacaine), applied locally to the surgical site, and 1/3 of a 50 μg/h/72 h fentanyl transdermal patch were used; in the following 5 days, analgesic (50 mg/kg/die) and antibiotic (10 mg/kg enrofloxacin) therapies were administered s. c. In the post-op, animals were clinically monitored daily in the first week and weekly thereafter, unless any complications occurred, by the control of the general conditions, weight measurements, major organ functions, and food and water consumption.

The following humane endpoints were set: loss of body weight greater than 20% of their physiological growth curve, severe limb lesions or irreversible alterations in major organic functions. Criteria for exclusion from analyses were severe intra-anaesthesiologic complications, knee infections or humane endpoint achievement.

#### 2.2.2 Surgical harvest of adipose tissue, tissue processing and IA treatments

Under general anaesthesia and aseptic conditions, inguinal adipose tissue (AT) was surgically collected and processed, as described in detail in a previous article (Desando et al., 2021), for i) the generation of *in vitro* expanded ASC and ii) the production of mctSVF. For the expanded-ASC, inguinal AT was harvested 2 weeks before IA treatments and treated with 0.4 U/mL NB4 collagenase standard grade (Serva Electrophoresis, GmbH, Heidelberg, Germany, Cat. DS17454.01) for 30 min at 37°C to obtain the SVF and then kept a fortnight until passage 2 in α-MEM (Gibco, Carlsbad, CA, United States) medium containing 15% fetal bovine serum (FBS, Euroclone) and 0.05 g/mL penicillin G (Gibco). Cell count was evaluated at SVF isolation and expansion.

As for mctSVF, the inguinal AT was harvested on the same day of IA injection and processed in a single sterile disposable device Hy-Tissue SVF (Fidia Farmaceutici, Abano Terme, Italy). After collection, AT was minced with scissors, resuspended with saline, and then moved into a closed sterile bag to perform mechanical fragmentation. Duografter (Fidia Farmaceutici) subjected the filtered intermediate adipose product to centrifugation at 600 g for 10 min to generate the final product (Desando et al., 2021).

Eight weeks after OA induction, animals received IA injection of 2 × 10^6^ ASC (n = 8) or mctSVF (n = 8) in each knee to assess their efficacy. For local biodistribution groups, 2 ×10^6^ ASC and mctSVF were labelled with 2 μM PKH26 (Sigma-Aldrich, Cat. PKH26GL), and injected in each knee to monitor cell migration at 1 month from treatments. Live&Dead test (Thermo Fisher Scientific, Waltham, MA, United States, Cat. L10119) was performed on all adipose products for efficacy analyses before the IA treatment. Live (green staining) and dead (red staining) cells were evaluated with the DS-Ri2 microscope (Nikon, Tokyo, Japan), by using fluorescence channels: fluorescein isothiocyanate (FITC) and tetramethyl rhodamine isothiocyanate (TRITC). Trypan blue staining was used to assess the number of viable labelled cells in the cell suspension before IA injection for biodistribution analysis. The dye stains non-viable cells in blue, while it is excluded from living cells.

#### 2.2.3 Experimental times and tissue explants

At the selected experimental times, rabbits were pharmacologically euthanized under deep general anaesthesia with intravenous administration of 1 mL of Tanax^®^ (Hoechst AG, Frankfurt-am-Main, Germany). Animals allocated in the local biodistribution groups were euthanized after 1 month from IA treatments: femoral condyles, tibial plateaus, menisci, synovial fluids and membranes and posterior cruciate and collateral ligaments were harvested for analysis through the assessment of the fluorescent cells with the DS-Ri2 microscope (Nikon, Tokyo, Japan). Briefly, biopsies were fixed with 10% neutral buffered formalin, decalcified when the bone component was present, paraffin-embedded, and microtome-cut. Dried cytospin preparations of synovial fluid were prepared after centrifugation for 10′ at 600 g in a cytocentrifuge (Thermo Fisher Scientific, Waltham, MA, United States). Then, cell nuclei of all samples were counterstained with 1 μg/mL 4′,6-diamidino-2-phenylindole (DAPI) (Sigma-Aldrich). Image analysis was performed with the NIS-Elements software, using the Hue/Saturation/Intensity (HSI) system, to quantify the percentage of positive cells at different specimen depths to better understand cell penetration within the tissues. Specifically, we presented a percentage of the stained cells over the total cells per tissue.

Animals allocated in the efficacy treatment groups were euthanized after 2 months from IA treatments: femoral condyles, meniscus and synovial membranes were explanted and processed for analyses.

### 2.3 Treatment efficacy on tissue samples: macroscopic and immunohistochemical analyses

Several methodologies were used to test the bio-functional properties of the osteochondral unit in the medial femoral condyle, synovial membrane and menisci in the control and experimental groups by two blinded investigators to the groups (GD and AC), according to the specific scores.

#### 2.3.1 Macroscopic assessment on articular cartilage

Outerbridge score was used for macroscopic analysis of femoral condyles in the control and experimental groups. This method allows a first assessment of articular cartilage, discriminating early, mild and severe degrees of OA. Specifically, the Outerbridge score has a value from 0 (an indicator of healthy tissue) to 4 (severe OA grade) (Laverty et al., 2010).

#### 2.3.2 Histological assessment on osteochondral, synovium and meniscus specimens

Next, histological examinations on different joint tissues were made to assess their architecture to ensure adequate function, using specific stainings with Safranin O/Fast green (Sigma Aldrich) for articular cartilage and meniscus and haematoxylin/eosin for synovial membrane. In general, seven sagittal sections per tissue, spaced 10 sections apart, were assessed to investigate the impact of cell treatments on cartilage and meniscus repair.

Specifically, we have used the Laverty score to provide a quantitative measurement of cartilage repair after treatments. This score has values ranging from 0 (healthy tissue) to 24 (severe OA) (Laverty et al., 2010). Parameters of this score, commonly associated with OA changes, include: (i) safranin-O staining to evaluate proteoglycan content; (ii) cartilage structure to assess extracellular matrix composition; (iii) chondrocyte density; and (iv) cluster formation.

As for subchondral bone, we have examined tissue architecture examining cellularity and bone marrow spaces using a semiquantitative score, which has a range from 0 (an indicator of healthy tissue) to 3 (an indicator of severe OA) (Aho et al., 2017).

Synovial specimens were stained with Hematoxylin/Eosin (Sigma Aldrich) to estimate their architectural appearance with a semi-quantitative analysis with Laverty score (Laverty et al., 2010). This score considers different parameters of synoviopathy, such as proliferation, hypertrophy, and inflammatory status, which affect tissue function.

As for the meniscus, with a pivotal role in knee biomechanics, a modified Pauli’s score was included in the study to assess its histopathological features during OA, including tears. This score shows a range from 0 to 18, where 0 is an index of healthy tissue and 18 of severe OA (Chevrier et al., 2009; Pauli et al., 2011).

#### 2.3.3 Immunohistochemical analysis on cartilage, bone, synovial and meniscus specimens

Further in-depth assessments were carried out on articular cartilage and synovial membrane using indirect colourimetric immunohistochemistry (IHC) with a dedicated panel of markers. Biotin-streptavidin (4plus Universal AP Detection; Biocare Medical) and the alkaline phosphatase detection system (Fast Red Substrate Kit; Biocare Medical) were used on biological replicates from the control and experimental groups. As for the articular cartilage, we used the following antibodies mouse monoclonal collagen type I (COL1) (2 μg/mL) (Sigma-Aldrich), mouse monoclonal collagen type II (COL2) (2 μg/mL) (Hybridoma Bank, Department of Biological Sciences, University of Iowa City, IA), mouse monoclonal matrix metalloproteinase (MMP)-3 (5 μg/mL) (Chemicon, Temecula, CA).

As for the subchondral bone, we tested osteocalcin (OC) (2 μg/mL) (Thermo Fisher Scientific, Waltham, MA, United States; Catalog #MA1-20788).

As for the synovial membrane, the protein expression of mouse monoclonal RAM-11 (1.5 μg/mL) (Dako) (macrophages found in inflammatory atherosclerotic plaques in rabbits), mouse monoclonal CD-163 (1 μg/mL) (Abcam) (macrophages with a regenerative phenotype) and MMP-3 (5 μg/mL) (Chemicon, CA) was performed to evaluate macrophage subtypes and catabolic processes, respectively.

As for the meniscus specimens, the protein expression of mouse monoclonal collagen type I (COL1) (2 μg/mL) (Sigma-Aldrich) and mouse monoclonal matrix metalloproteinase (MMP)-3 (5 μg/mL) (Chemicon, CA) was carried out.

The selection of markers has been made based on the anabolic and catabolic processes that are involved in joint degeneration in OA. Before incubation with the primary antibodies, specific unmasking procedures were done for articular cartilage with 1 mg/mL pronase (Sigma-Aldrich). Negative controls were run by omitting the primary antibodies or using an isotype-matching control.

Image analysis was performed on specimens immunostained to provide a quantitative assessment. Specifically, the Hue Saturation Intensity (HSI) system was adopted to define the threshold of positivity for each marker and establish a percentage value of positivity, ranging from 0 (no positivity) to 100 (highest positivity). All assessments were conducted by trained scientists (AC, GD) using a DS-Ri2 microscope (Nikon, Tokyo, Japan), blind to the type of treatment.

### 2.4 Statistical analysis

The Statistical Package for the Social Sciences (SPSS Inc., Chicago, IL, United States) software version 15.0 (SPSS Inc.) was used for statistical analyses. The presence of outliers was checked using the ROUT test (Q = 1%) before each test. If present, outliers were removed from each dataset. Kolmogorov-Smirnov (K-S) and Shapiro-Wilk tests were carried out to test the normality of data distribution. Datasets showing a Gaussian distribution were assayed by one-way ANOVA and general linear model (GLM) tests with the Sidak *post hoc* test for multiple comparisons. Specifically, a one-way ANOVA and Sidak tests were used to assess differences in cell biodistribution between ASC and mctSVF groups, which showed a normal distribution. Datasets from macroscopic and immunohistochemical assessments, that showed a normal distribution, were assayed the GLM with Sidak correction for multiple comparisons. In particular, the GLM with Tweedie/gamma distribution and log link function was used to analyse the parameters comprising the histological scores of cartilage, synovium, and meniscus (mPauli); the total score was the dependent variable and the cases were the fixed effects.

Datasets with no Gaussian distribution, as observed for the number of nucleated cells and cell viability were assayed with the Kruskall-Wallis and Dunn’s *post hoc* test. The estimated expected values with Wald 95% confidence intervals were compared using contrasts versus OA and Sidak correction for multiple comparisons. The non-parametric Spearman’s rank-order method was used to find correlations between viable cells of ASC and mctSVF treatments and (1) cartilage score; (2) synovium score; and (3) meniscus score. Data were considered significant at *P* < 0.05 (*), *P* < 0.01 (**), *P* < 0.001 (***), and *P* < 0.0001 (****).

## 3 Results

### 3.1 Report on adverse effects

No fractures, infections or changes in major organ functions were found in the post-op period. Some minor side effects mainly related to the type of surgery and the experimental model were found; in particular, 7/32 animals showed moderate joint effusion on the first post-op day, which resolved in 3–4 days, 10/32 animals developed lameness that resolved 1 week after surgery and 2/32 animals received analgesics for further 72 h. All cases were included in the data analysis. As detailed in the figure legends, for the SHAM and OA groups, we included additional cases from previous studies, conducted using the same surgical protocols and procedures, to increase the statistical power according to the principles of the 3Rs.

### 3.2 The mechanical process with Hy-tissue SVF produces viable cells

**FIGURE 1:**
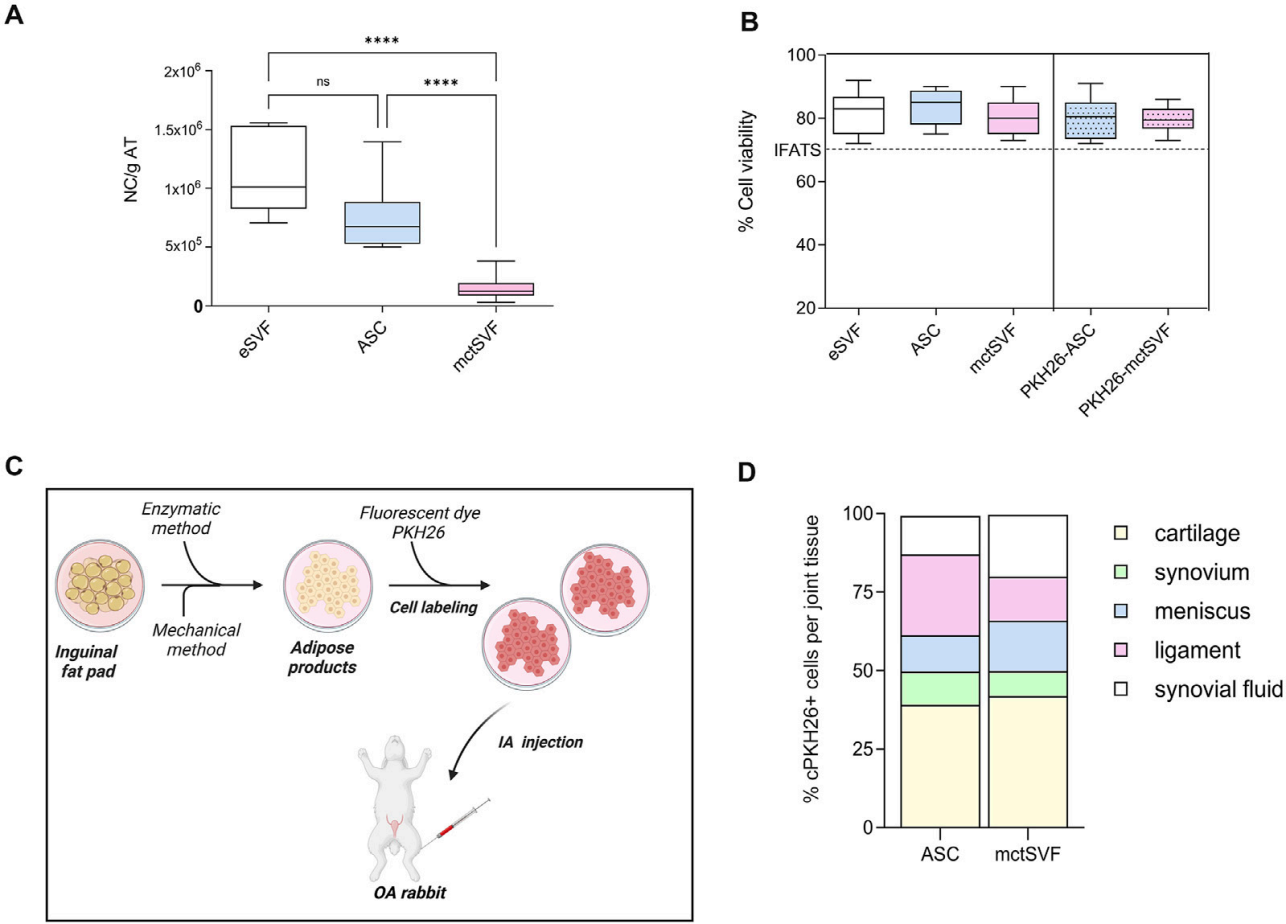
**(A)** Graphical representation of the number of nucleated cells (NC)/g of adipose tissue (AT) in enzymatic stromal vascular fraction (eSVF), ASC and mctSVF samples. **(B)** Graph of the percentage of cell viability, reported as mean, standard deviation and 95% confidence intervals, in eSVF, ASC, mctSVF after AT processing and PKH26-ASC and PKH26-mctSVF after labelling for local biodistribution assessment. The dotted line shows the 70% threshold suggested by the IFATS to consider a cell treatment clinically eligible. Results from the Kruskall-Wallis test and Dunn’s test are reported (n = 8 samples/group). ****P < 0.0001 NC of ASC versus mctSVF; ****P < 0.0001 NC of eSVF versus mctSVF. **(C)** Schematic workflow of *in vitro* labelling of ASC and mctSVF before IA treatment with Biorender software http://Biorender.com/q879319. **(D)** Graphical representation of the percentage of local distribution of ASC (n = 8 articular joints: right and left hind limbs) and mctSVF (n = 8 articular joints: right and left hind limbs) in the entire articular joint. Percentage of cell positivity in synovial fluids, ligaments, menisci, synovia and articular cartilage at 1-month follow-up from the injection.

As a quality control, mctSVF cells were counted with the Turk stain and tested using the Live&Dead assay to monitor cell number and viability, respectively. The interval range of the mctSVF cells produced was from 3.5 × 10^4^ to 3.5 × 10^5^ nucleated cells/g of adipose tissue. Conversely, eSVF and ASC showed a higher amount of nucleated cells/g of adipose tissue, a mean value of 1.15 × 10^6^ and 7.3 × 10^5^, respectively (Figure 1A). Comparable values of cell viability among enzymatic SVF, expanded ASC and mctSVF were found, by reporting optimal percentages. The PKH26 staining had no effects on cell viability. According to the guidelines proposed by the International Federation for Adipose Therapeutics and Science (IFATS), all cell products showed good potential for clinical translation (Figure 1B) (Desando et al., 2021). We found a negative correlation between the cell viability of both cell groups and the cartilage, synovial, and meniscus scores (Supplementary Table 2). The highest levels of cell viability correlated with the lowest scores, reflecting better tissue repair. This suggests that higher levels of cell viability are associated with improved histological outcomes.

### 3.3 PKH26-ASC and mctSVF accumulate to a higher level in the cartilage at 1 month

Analysis of cell migration using a fluorescent dye, PKH26, was performed to track the distribution of ASC and mctSVF products to provide some mechanistic insights on their potential clinical translation in OA (Figures 1C, D). PKH26-ASC and PKH26-mctSVF showed a high tropism towards injured areas of articular cartilage (cell clones and fibrillated areas) after 1 month. In the ASC group, the cartilage tissue showed the highest percentage of labelled cells, displaying an increased percentage of 29.3%, 28.3%, 14.3%, and 27.7% than synovia, meniscus, ligaments, and synovial fluids, respectively. Both synovia and meniscus also showed the lowest migration pattern of 15% and 14% than the ligaments. ASC group reported a significantly higher percentage of cell distribution in cartilage than in the meniscus and ligaments. Similarly to ASC, the mctSVF group showed most cells migrating towards cartilage reporting higher values of the distribution pattern of 34%, 25.8%, 23%, and 26.7% than synovia, meniscus, ligaments, and synovial fluids, respectively. Labelled mctSVF showed a minor tropism to the synovial membrane, which displayed also reduced values than the meniscus. mctSVF group showed significantly higher mean values of distribution in cartilage than the meniscus and ligaments. Significant differences in the migration pattern between the two cell-treated groups were found only at the meniscal level, with a higher distribution pattern for mctSVF than the ASC group (Figure 1C) (Supplementary Tables 3, 4).

### 3.4 mctSVF treatment protects the cartilage from OA-related structural degradation

**FIGURE 2:**
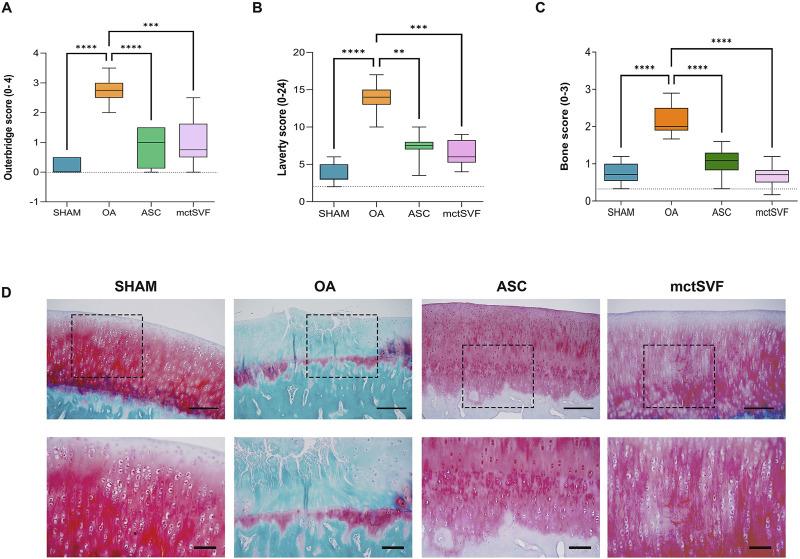
Graphical representation of Outerbridge score **(A)**, Laverty’s score **(B)** and bone score **(C)**, reported as mean, standard deviation and 95% confidence intervals. **(D)** Representative histological images of medial femoral condyle with Safranin-O/Fast green staining in SHAM (n = 11), OA (n = 13), ASC (n = 8) and mctSVF (n = 8) groups at low (scale bar = 100 μm) (upper panel) and high magnification (scale bar = 50 μm) (lower panel). Red/pinkish staining: proteoglycan content; green staining: collagen content. The general linear model (GLM) with Sidak correction for multiple comparisons was used to assess the efficacy of ASC and mctSVF on all joint tissues. Outerbridge score: ****P < 0.0001 ASC vs. OA; ***P < 0.001 mctSVF vs. OA. Laverty’s score: **P < 0.01 ASC vs. OA; ***P < 0.001 mctSVF vs. OA. Bone score: ****P < 0.0001 ASC vs. OA, and mctSVF vs. OA.

**FIGURE 3:**
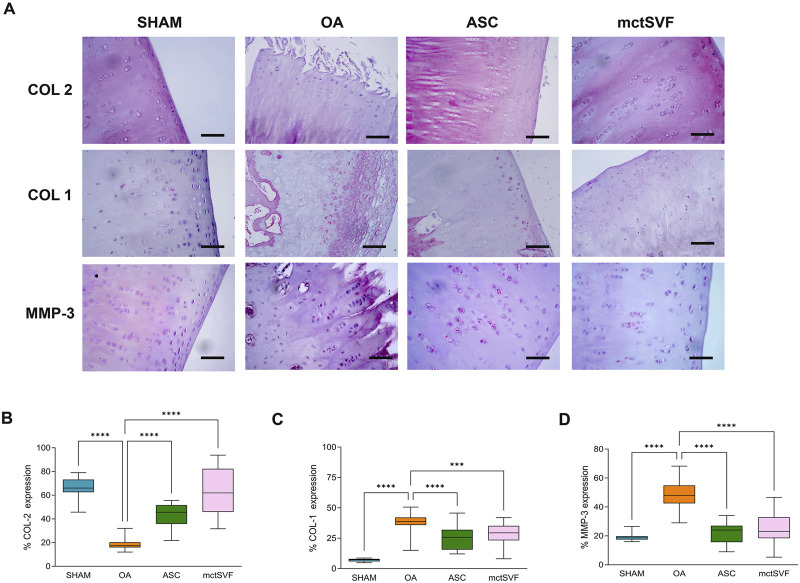
Representative immunohistochemical images of the medial femoral condyle in SHAM, OA, ASC and mctSVF-treated groups for COL2, COL1 and MMP-3 **(A)** (scale bar = 50 μm). Graphical representation of image analysis expressed as the percentage of positivity for COL2 **(B)**, COL1 **(C)** and MMP-3 **(D)**, reported as mean, standard deviation and 95% confidence intervals, in SHAM (n = 11), OA (n = 13), ASC (n = 8) and mctSVF (n = 8) groups. The general linear model (GLM) with Sidak correction for multiple comparisons was used to assess the efficacy of ASC and mctSVF for cartilage repair. COL2: ****P < 0.0001 ASC vs. OA and mctSVF vs. OA. COL1: ****P < 0.0001 ASC vs. OA; ***P < 0.001 mctSVF vs. OA. MMP-3: ****P < 0.0001 ASC vs. OA and mctSVF vs. OA.

After 8 weeks from ACLT surgery, the femoral condyles of the OA group were analyzed to evaluate histopathological features (Figures 2A–D). The macroscopic assessment showed fibrillation areas and the presence of greater erosive zones in the medial femur. The IA treatment with ASC and mctSVF provided several benefits in reducing macroscopic OA features such as fibrillation and erosion in the articular cartilage. In particular, the Outerbridge score, a semi-quantitative measure of the macroscopic OA severity process, demonstrated a significant OA reduction especially after ASC-based therapy and to a lesser extent in the mtcSVF group (Figure 2A). As for the OA group, histochemical staining with Safranin-O/Fast green confirmed macroscopic assessments showing a rough articular surface, reduced articular thickness, irregular cell organization interspersing cell clones and empty areas, and reduced proteoglycan content (Figure 2B). On a 2-month follow-up after ASC and mctSVF treatments, several repair processes were evident in the femur which displayed a regular surface, proper cell distribution and density, and adequate proteoglycan content, similar to the SHAM group. Histochemical semi-quantification of the OA changes with Laverty’s score showed a great ability of both cell therapies to counteract OA-related structural degradation (restoration of cell distribution and matrix organization), with a significantly lower score than the OA group (Figure 2B). Specifically, cartilage features related to safranin-o content, structure and cell clones, which are some of Laverty’s parameters, were noticeably improved in both cell treatments. However, a significant improvement in cell density was only observed following the treatment with mctSVF (Supplementary Figure 4). IHC analyses showed the lowest percentage of COL2 positivity in the OA group, mainly confined in the deep cartilage layer, near the tidemark. Both ASC and mctSVF treatments contributed to a significantly increased positivity of this marker when compared to the OA group, mainly diffused in the extracellular matrix (ECM) in the superficial and the deep cartilage. (Figures 3A, B). Moreover, COL1 and MMP-3, specific markers associated with cartilage degeneration, were assessed (Figure 3). COL1 protein expression was highly observed at the cellular level in the OA group than in the SHAM group (Figures 3A, C). A significant reduction of COL1 expression was mainly observed after the IA treatment with ASC and mctSVF. As for MMP-3, the OA group displayed a strong cellular positivity mainly close to cell clones and fibrillated areas. The IA treatment with both ASC and mctSVF significantly reduced its expression by showing similar values to the SHAM group (Figures 3A, D).

### 3.5 IA administration of mctSVF improves subchondral bone architecture similar to ASC

**FIGURE 4:**
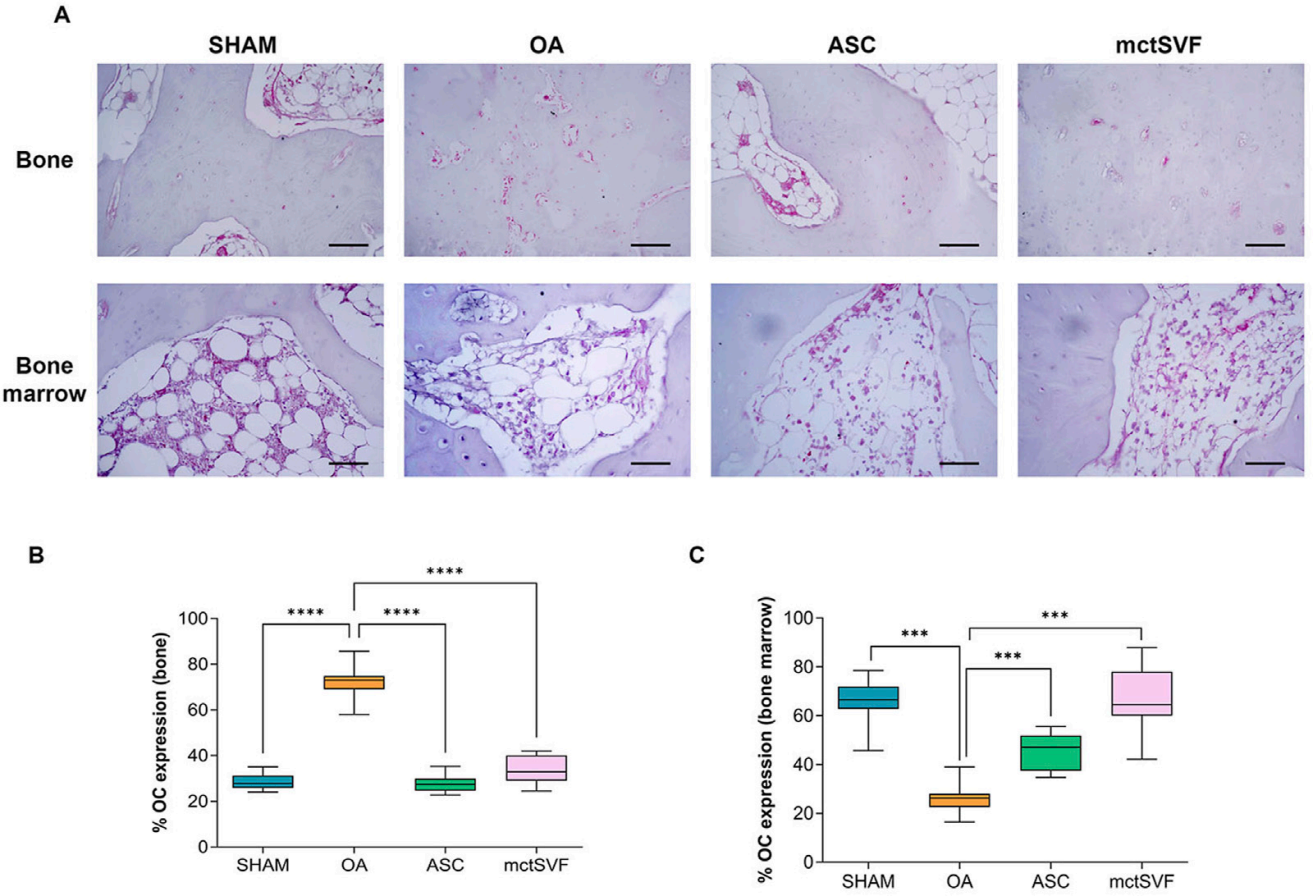
Representative immunohistochemical images of subchondral bone in medial femoral condyle in SHAM, OA, ASC and mctSVF-treated groups for osteocalcin (OC) in the bone matrix and medullary cavity (scale bar = 50 μm) **(A)**. Graphical representation of image analysis expressed as the percentage of positivity for OC in bone matrix **(B)** and medullary cavity **(C)**, reported as mean, standard deviation and 95% confidence intervals, in SHAM (n = 11), OA (n = 13), ASC (n = 8) and mctSVF (n = 8) groups. The general linear model (GLM) with Sidak correction for multiple comparisons was used to assess the efficacy of ASC and mctSVF for bone repair. OC (bone): ****P < 0.0001 ASC vs. OA and mctSVF vs. OA. OC (bone marrow): ***P < 0.001 ASC vs. OA and mctSVF vs. OA.

To better understand the biological changes in the knee joint and how treatments differentially modulate them, specific IHC studies were performed in detail on trabecular bone and bone marrow. The baseline condition observed in the untreated group depicts a histological picture characterized by altered tidemark integrity, subchondral bone thickness, reduced number of trabecular bone and medullary spaces rich in adipose tissue, and low cell counts. Qualitative histological analysis of the ASC and mctSVF groups showed some regenerative processes, as evidenced by an increase in the number of trabecular bone and the cell number within the bone marrow spaces, similar to the SHAM group, with a low number of osteoclasts, identified with haematoxylin/eosin staining for their typical multinucleated features. The scoring system used for examining the bone characteristics gave evidence of a better architecture in both treated groups reporting a significantly lower score, index of a better bone structure, than the OA group (Figure 2C). IHC analysis for the OC marker, one of the most abundant non-collagenous proteins in the bone matrix (Desando et al., 2021; Rodrigues et al., 2012), was examined to further assess bone repair after the IA treatments (Figure 4). The untreated OA and experimental groups showed different patterns of positivity for this marker between the medullary cavity and the bone compartment. Interestingly, the OA group showed positive staining in cells embedded in the bone matrix and located in the medullary cavities, whereas treated groups had mainly a marked positivity in the medullary cavities than the bone matrix (Figures 4A–C). Image analysis showed a significantly higher level of OC protein expression in the medullary cavities in both ASC/mctSVF treatments than in the OA group, and similar values to the SHAM group.

### 3.6 IA administration of ASC and mctSVF improves synovial architecture

**FIGURE 5:**
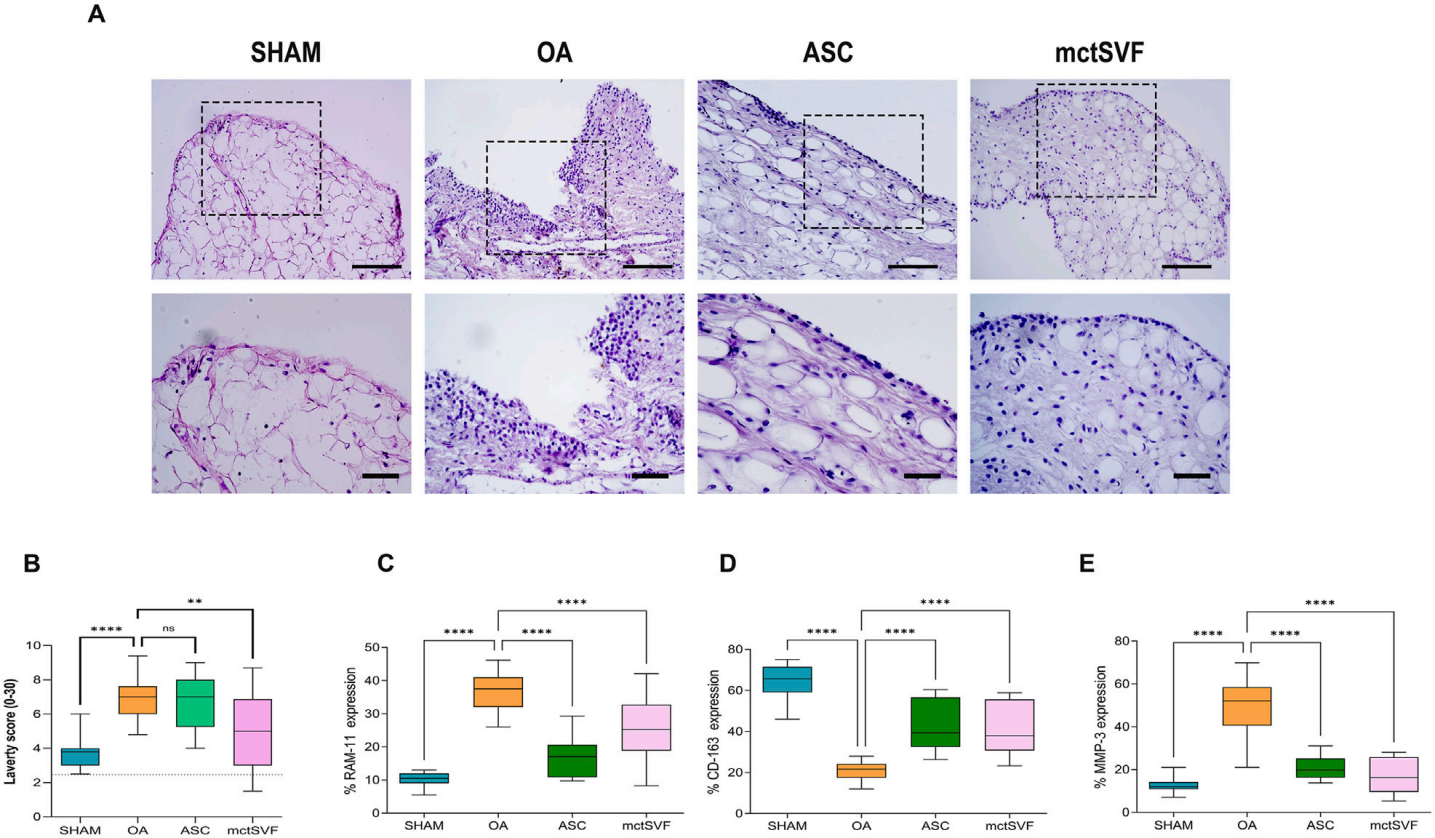
Representative histological images of the synovial membrane with hematoxylin/eosin staining in SHAM, OA, ASC and mctSVF groups at low (bar = 100 μm) (upper panel) and high magnification (lower panel) (scale bar = 50 μm) **(A)**. Graphical representation of Laverty’s score **(B)** and image analysis for RAM-11 **(C)**, CD-163 **(D)** and MMP-3 **(E)**. in the synovial membrane reported as mean, standard deviation and 95% confidence intervals in SHAM (n = 11), OA (n = 13), ASC (n = 8) and mctSVF (n = 8) groups. The general linear model (GLM) with Sidak correction for multiple comparisons was used to study the impact of ASC and mctSVF on synovium tissue. Laverty’s score: **P < 0.01 mctSVF vs. OA. RAM-11, CD-163 and MMP-3: ****P < 0.0001 ASC vs. OA and mctSVF vs. OA.

Further analyses on the synovial architecture and synovial macrophage activity were performed to assess how they may be modulated after ASC and mctSVF treatments and how they may impact tissue repair in our ACLT model. As for the OA group, qualitative analyses with H/E staining showed hypertrophic and hyperplastic processes in the synovial lining layer, resulting in synovial thickness. In addition, some degenerative aspects with a vascular ECM and some inflammatory infiltrates were also observed in this group. Conversely, both ASC and mctSVF groups displayed a smooth surface with decreased hypertrophy and vascularization in the sub-synovium and a substantial lowering of OA characteristics. The semi-quantification with Laverty’s score confirmed some benefits of both cell-based treatments, with significant evidence only from the mctSVF group (Figures 5A, B) (Supplementary Figure 2). Further analyses were focused on the evaluation of some markers beyond tissue catabolism and macrophage activation by IHC. As for the macrophage component, both treatments lowered the protein expression of RAM-11 in cells located in the lining layer, typically expressed by the inflammatory atherosclerotic plaques in rabbits. Conversely, both treatments increased protein expression for the CD-163 marker, typical of M2c-like macrophages (Figures 5C, D). The catabolic mediator MMP-3 was at its highest level in the OA group and mainly located in the lining layer. Interestingly, showing a similar pattern to the SHAM group, the IA delivery of both ASC and mctSVF significantly downregulated MMP-3 expression compared to the OA group (Figure 5E) (Supplementary Figure 3).

### 3.7 ASC and mctSVF contribute to lower typical features of meniscus degeneration

**FIGURE 6:**
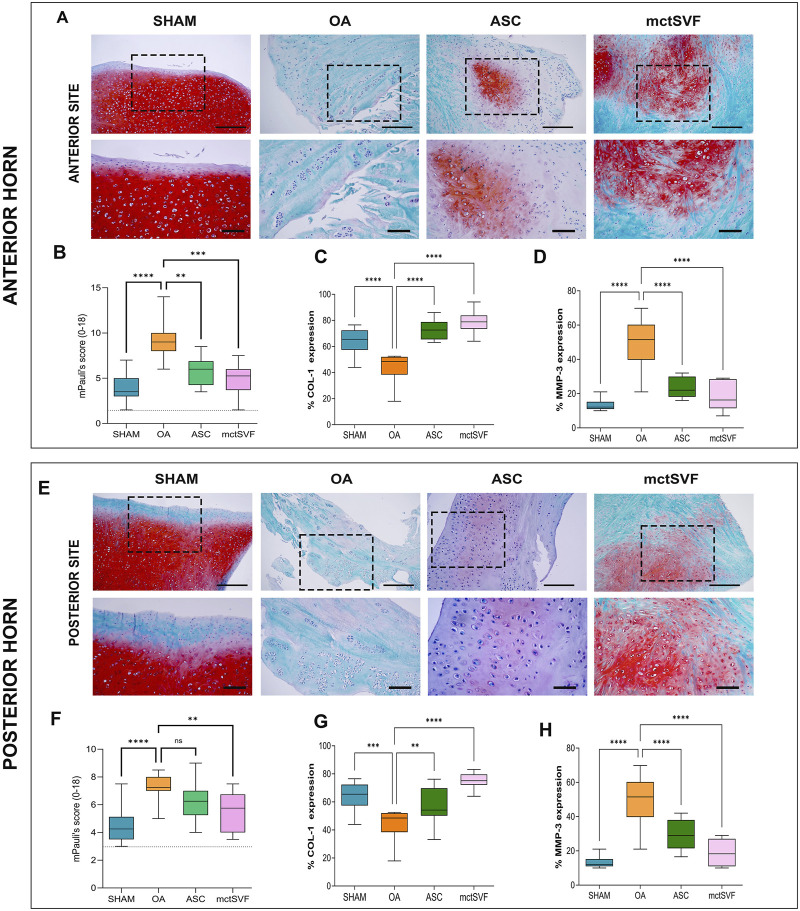
**(A)** Representative histological images of the medial meniscus with Safranin-O/Fast green in SHAM, OA, ASC and mctSVF groups at low (scale bar = 100 μm) and high magnification (scale bar = 50 μm) in the anterior horn (upper panel). **(B–D)** Graphical representation of mPauli’s score, COL-1 and MMP-3 analyses in the anterior horn of the medial meniscus, reported as mean, standard deviation and 95% confidence intervals SHAM (n = 11), OA (n = 13), ASC (n = 8) and mctSVF (n = 8) groups. The general linear model (GLM) with Sidak correction for multiple comparisons was used to study the impact of ASC and mctSVF in the anterior horn of the medial meniscus. mPauli’s score: ****P < 0.0001 SHAM vs. OA, **P < 0.01 ASC vs. OA, ***P < 0.001 mctSVF vs. OA; COL-1: ****P < 0.0001 SHAM vs. OA, ****P < 0.0001 ASC vs. OA; ****P < 0.0001 mctSVF vs. OA. **(E)** Representative histological images of the medial meniscus with Safranin-O/Fast green in SHAM, OA, ASC and mctSVF groups at low (scale bar = 100 μm) and high magnification (scale bar = 50 μm) in the posterior horn (lower panel). **(F–H)** Graphical representation of mPauli’s score, COL-1 and MMP-3 analyses in the posterior horn of the medial meniscus, reported as mean, standard deviation and 95% confidence intervals SHAM (n = 11), OA (n = 13), ASC (n = 8) and mctSVF (n = 8) groups. The general linear model (GLM) with Sidak correction for multiple comparisons was used to study the impact of ASC and mctSVF on the posterior horn of the meniscus: mPauli’s score: **P < 0.01 mctSVF vs. OA; COL-1: ***P < 0.001 SHAM vs. OA, **P < 0.01 ASC vs. OA, ****P < 0.0001 mctSVF vs. OA; MMP-3: ****P < 0.0001 SHAM vs. OA, ****P < 0.0001 ASC vs. OA, ****P < 0.0001 mctSVF vs. OA.

Given that meniscal lesions can lead to knee biomechanical abnormalities with important implications for OA progression, specific histological evaluations have been performed for both anterior and posterior anatomical sites of the meniscus. During the qualitative analysis using Safranin-O/Fast Green staining, we observed an increase in tears, cell clones, and proteoglycans in the OA group in both the anterior and posterior zones. In contrast, both ASC and mtcSVF treatments played an active role in counteracting OA-related structural degradation through (i) the reduction of tears and cell clones, and (ii) the restoration of proteoglycan content and cell distribution in the ECM. Semi-quantitative analysis of histochemical OA changes with mPauli’s score showed a worse histological scenario in the anterior rather than the posterior horn of the medial meniscus in the OA group. Interestingly, a significantly lower mPauli’s score, an index of tissue repair, was observed following ASC and mtcSVF treatments compared to the OA group; however, no differences were found between the two treatments (Figure 6) (Supplementary Figures 4, 5). Immunohistochemical analyses confirmed regenerative processes after ASC and mctSVF therapies, reporting an increase of COL-1 and a reduction of MMP-3 in both the anterior and posterior horns of the medial meniscus (Figure 6).

## 4 Discussion

The search for alternative options to halt or delay the progression of OA and consequently joint replacement surgery has sparked great interest. Within the musculoskeletal field, an emerging trend is the development of biological approaches that process and maintain 3D tissue interactions as an alternative to other cell-based solutions. Point-of-care (POC) systems require minimal manipulation of biological samples to preserve the biophysical and biochemical cues of the native tissue microenvironment (Oberbauer et al., 2015; Greenwood et al., 2022; Aronowitz, 2014). With a focus on adipose-derived therapies (Ossendorff et al., 2023; Agarwal et al., 2021), this preclinical *in vivo* study aims to provide new bio-insights into the orthobiologic product mctSVF, obtained by the Hy-Tissue SVF system (Fidia Farmaceutici, Abano Terme) for OA treatment, compared to the ASC-based therapy (Agarwal et al., 2021). A preliminary *in vitro* study indicated that wrapping mctSVF in its native microenvironment ensures optimal cell viability and preserves phenotypic and functional properties (Desando et al., 2021). It has been proposed that the potential use of mctSVF in the treatment of OA may be explained by its ability to remain functionally active due to its spatial distribution and cell-matrix interaction. In particular, it was hypothesized that the IA delivery of mctSVF can exhibit greater stability at a joint injury site and superior ability than ASC to attach to and penetrate joint tissue. This is based on the notion that the maintenance of the native tissue structure within mctSVF is crucial to preserve the stemness of tissue and to uphold native crosstalk with complex and multivalent signals (Gattazzo et al., 2014; Hynes, 2009). Given the multifaceted and multicompartmental nature of the OA phenotype (Mobasheri et al., 2019), various biological processes (inflammation, degradation, hypertrophy, and regeneration) need to be sufficiently characterized to assess the successful translation of mctSVF in clinics.

First, in line with the majority of studies on different SVF formulations (Boada-Pladellorens et al., 2022), this work highlighted the lack of adverse events and the efficacy of the current mctSVF micrograft product to improve preclinical outcomes in a post-traumatic OA model, similar to ASC. Interestingly, we found a correlation between cell viability and scoring systems, leading to the mechanistic conclusion that cell viability plays a relevant role in promoting tissue repair by ensuring a sufficient number of effective cells. IA delivery of ASC and mctSVF promoted comparable cell stabilization in the articular cartilage surface (Huang et al., 2022). We can speculate that similar cell migration may be the result of matrix degradation of mctSVF by enzymes in the *in vivo* microenvironment at 30 days, with the release of single cells, as occurs for ASC treatment. The lack of a short-term study, which is a limitation in evaluating local biodistribution, precludes providing information on the fate of cells following IA administration and early initiating events responsible for joint repair. This may help to better understand the mechanisms that go beyond tissue repair and to elucidate which are the first tissues to be involved in remodelling. Indeed, the lack of adverse effects on joint tissues and the cell engraftment observed at 2 months mainly to damaged areas of articular cartilage would indicate a tendency to promote cartilage repair by counteracting OA progression. To a lesser extent, the presence of cells in the adjacent joint tissues like the synovial membrane and meniscus provides indications of their potential role in modulating tissue remodelling in the articular joint. Moreover, the long-term assessment gives clues about cell integration into the articular cartilage, which may reflect the observed chondroprotective effects, similar to another study (Huang et al., 2022).

Second, the current study confirmed the repair potential of mctSVF to restore the hierarchical structure and functionality of the osteochondral tissue, crucial for facilitating metabolic exchange and joint biomechanics and restoring proper cellularity (Nukavarapu and Dorcemus, 2013). Although the cell yield of the mechanical procedure with Hy-tissue SVF is much lower than the enzymatic isolation, the tissue repair of mctSVF can have different interpretations. In particular, it may likely be the direct result of: (i) ASC (representing up to 10%), and (ii) cell complexes within its adipose-connective niche (ACN). ASC and ACN, the latter with a heterogenous reservoir of cells, could be active instructive players by: (1) fostering osteogenic and chondrogenic differentiation; and (2) modulating inflammatory and regenerative processes in the articular microenvironment thanks to their paracrine activity. A previous *in vitro* study demonstrated the osteochondral potential of this micrograft and the release of some biomolecules, like VEGF and HGF, that may have clinical benefits in the osteochondral microenvironment (Desando et al., 2021; Traktuev et al., 2009). Despite the promising findings on such biomolecules, further studies aimed at investigating mechanisms beyond bone repair will require careful analysis of markers involved in bone resorption and deposition.

There has been a growing body of knowledge on the clinical significance of the correct interplay between bone and cartilage to protect cartilage, with bone nourishing articular cartilage and protecting it against degradative changes such as proteoglycan depletion (Nukavarapu and Dorcemus, 2013). Additionally, the enhanced proteoglycan content observed in the ECM of articular cartilage following cell-based treatments may be attributed to the secretion of HGF, reported *in vitro* for both ASC and mctSVF (Desando et al., 2021; Pak et al., 2014).

A major hurdle in cartilage repair, and the reason most treatments fail, is the formation of fibrocartilage. In particular, COL1 in articular cartilage is an indicator of ineffective cartilage repair, as the newly formed tissue, due to its fibrous profile, is unable to provide the correct biomechanical properties and joint loading. This study gives collective evidence towards the benefits of mctSVF treatment in counteracting fibrocartilage formation in favour of a hyalin-like phenotype, similar to ASC treatment. Similar to ASC, the mctSVF treatment effectively counteracts MMP-3 protein expression, a typical protease involved in COL2 cleavage. This is a further indication of its potential therapeutic value in inhibiting the reduction of this typical cartilage marker, which is often at low levels in OA conditions.

Third, tissue regeneration processes were also observed in the meniscus following ASC and mctSVF treatments, consistent with other studies focusing on this cell approach (Pak et al., 2014). Although at a low rate, the local biodistribution of ASC, and especially of mctSVF in the meniscus, could contribute to the reduction of several degenerative changes such as tearing (Pak et al., 2014). However, understanding this behaviour would require further analysis, which could open new insight into different orthopaedic contexts where meniscus lesions occur.

Fourth, attenuating the immune response at the injection site through immunomodulation may help to regenerate the host tissue. As natural components of the immune microenvironment of the joint, synovial macrophages are known to adopt either pathogenic or protective phenotypes in response to microenvironmental stimuli (Chen et al., 2020), and their role in OA pathogenesis is well established. By evaluating the macrophage profile in synovial samples after treatment, we found that both mctSVF and ASC effectively reduced fibrosis in terms of reduction of COL1 and drove the synovial macrophage subset towards a CD-163 phenotype, expressed by the M2 macrophage subset. Indeed, the release of interleukin (IL)-10 found in a previous *in vitro* study characterizing mctSVF could reflect the change of macrophage phenotype from an inflammatory to a regenerative profile. However, to clarify this aspect and provide new insights into the role of potential immunomodulatory strategies for cartilage regeneration in OA (Kennedy et al., 2024), further research is needed. Specifically, modelling specific *in vitro* human culture systems with immunomodulatory molecules, such as anti-programmed death ligand (PDL1), an immune checkpoint whose blockade causes the development of OA in mice (Liu et al., 2019), might be a promising alternative to provide interesting insights in immune response attenuation.

A further suggestion for the mechanism of action underlying the use of ASC and mctSCF is the reduced expression of serum IL-10 and tumour necrosis factor (TNF)-α, which is characteristic of patients with post-traumatic OA, in favour of an inflammatory phenotype (Barker et al., 2021). The high level of IL-10 expression found in previous *in vitro* studies for the adipose cell-based strategies analyzed could re-establish an appropriate IL-10/TNF-α ratio, which is necessary to counteract some of the typical hallmarks of OA (i.e., inflammation, hypertrophy, fibrosis, etc.). Valuable insights into the factors and pathways involved in tissue regeneration could be gained from further *in vitro* studies modelling co-cultures and three-dimensional models between mctSVF and typical joint cells such as chondrocytes, osteoblasts and synoviocytes.

The lack of a comparative study between enzymatic and mechanical SVF using only expanded-ASC as a comparator group is a limitation of this study. Evidence for the safety and efficacy of enzymatic SVF as an injective intervention or augmentation of surgical procedures is available from various preclinical and clinical studies. However, they are often clinical case series with heterogeneous experimental designs. In general, it has been observed that the SVF has many advantages over the ASC being: 1) immediately available without the need for cell culture; 2) immediately available thanks to faster processing and direct delivery on the same day of the surgery. Mechanical processing alternatives have been proposed to overcome some of the concerns associated with enzymatic treatment. Although mechanical processing often provides a lower number of available cells, it ensures comparable and/or superior chondroprotective and immunomodulatory properties (Aletto et al., 2022).

Given these findings, tissue manipulation with the Hy-Tissue SVF system preserved the phenotypical and functional characteristics of the generated mctSVF without altering its regenerative potential, thus exerting disease-modifying effects on the joint tissue. Because of the similar profile of effectiveness between the 2 cell therapies, it would be more convenient to use mctSVF treatment by avoiding issues related to cell manipulation (enzymatic isolation, cell expansion, etc.) and the need for a GMP facility, by minimizing the costs. Indeed, a major clinical limitation of the mctSVF approach is the variability of cell precursors and bioactive molecules within the ACN, which does not guarantee similar clinical outcomes among OA patients. The patient’s age, comorbidities (obesity, diabetes, etc.), lifestyle, site of aspiration of adipose tissue, and quality of adipose tissue are other key variables contributing to the variability in SVF yield and cell potency. In this context, a thorough evaluation of the harvested tissue’s viability covers a relevant role in fostering tissue repair by ensuring an adequate amount of effective cells *in vivo*. Indeed, the development of standardized protocols for harvesting and processing SVF are another critical step, that might contribute to reducing the variability in SVF efficiency (Jeyaraman et al., 2024). In-depth studies aimed at evaluating a cohort of patients, considering age, gender, body mass index, and inflammation, may be a valuable tool for selecting patient groups having more advantageous benefits to receive this treatment, and to develop more personalized treatments.

Taken together, our results demonstrate that IA delivery of mctSVF is safe, effective and minimally invasive, accelerating cartilage repair and shifting the balance from cartilage destruction to chondroprotection with potential translatability to different orthopaedic clinical conditions.

## Data Availability

The original contributions presented in the study are included in the article/Supplementary Material, further inquiries can be directed to the corresponding author.
