# Supplementary material for: 3D connective micro-fragment enriched with stromal vascular fraction in osteoarthritis: chondroprotective evidence in a preclinical in vivo model
Source: Front Cell Dev Biol. 2025 Feb 27;13:1533405. doi: 10.3389/fcell.2025.1533405 (PMC11903414; doi:10.3389/fcell.2025.1533405)
Supplement: Supplementary file 1 [file DataSheet2.pdf]

| Variables | Spearman correlation between cartilage score and variables | | Spearman correlation between synovial score and variables | | Spearman correlation between the score of the anterior horn of the medial meniscus and variables | | Spearman correlation between score of the posterior horn of medial meniscus and variables | |
|---|---|---|---|---|---|---|---|---|
| | *P* value | Rho value | *P* value | Rho value | *P* value | Rho value | *P* value | Rho value |
| ASC viability | $^{*}P < 0.05$ | -0.95 | $^{*}P < 0.05$ | -0.97 | $^{**}P < 0.01$ | -0.98 | $^{*}P < 0.05$ | -0.95 |
| mctSVF viability | $^{***}P < 0.001$ | -0.95 | $^{***}P < 0.001$ | -0.83 | $^{**}P < 0.01$ | -0.79 | $^{*}P < 0.05$ | -0.85 |

**Supplementary Table 2**. Relationships between cell viability and tissue scores (cartilage, synovial and meniscus scores) for ASC and mctSVF groups. $^{*}P$ values < 0.05 were considered significant.
