# Supplementary figures and images for: 3D connective micro-fragment enriched with stromal vascular fraction in osteoarthritis: chondroprotective evidence in a preclinical in vivo model
Source: Front Cell Dev Biol. 2025 Feb 27;13:1533405. doi: 10.3389/fcell.2025.1533405 (PMC11903414; doi:10.3389/fcell.2025.1533405)

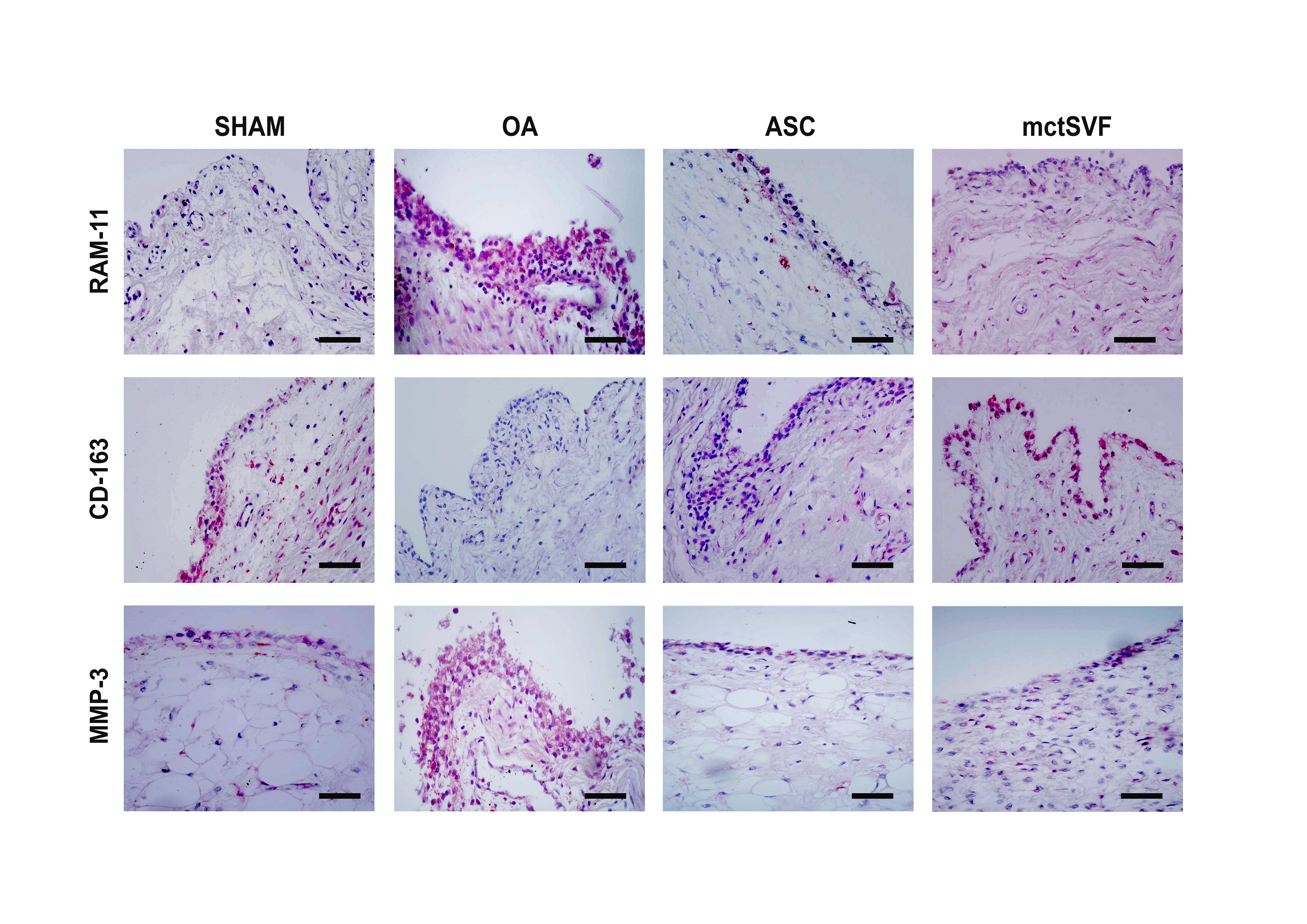

Supplement: Supplementary file 2 [file Image3.jpeg]

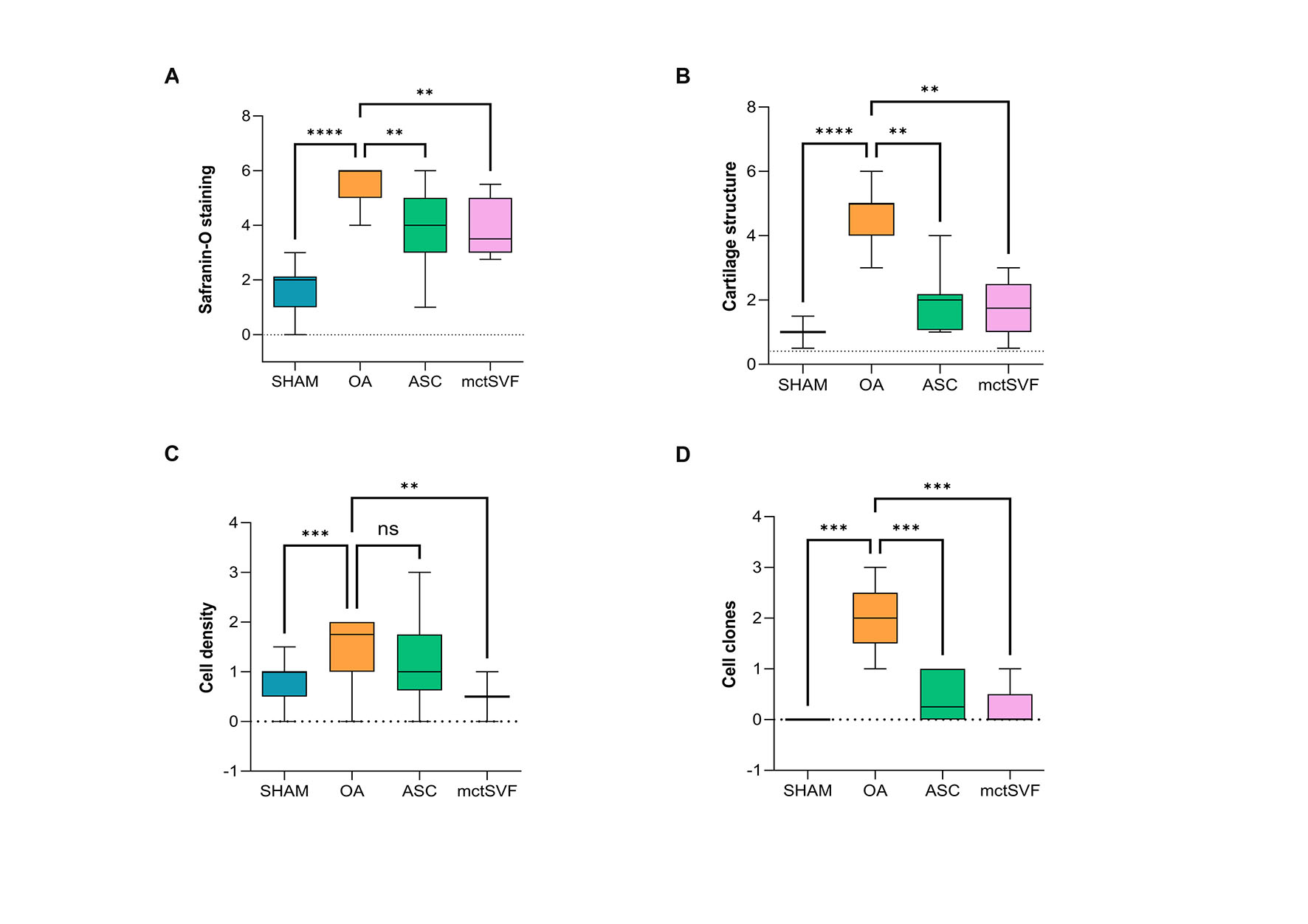

Supplement: Supplementary file 4 [file Image1.jpeg]

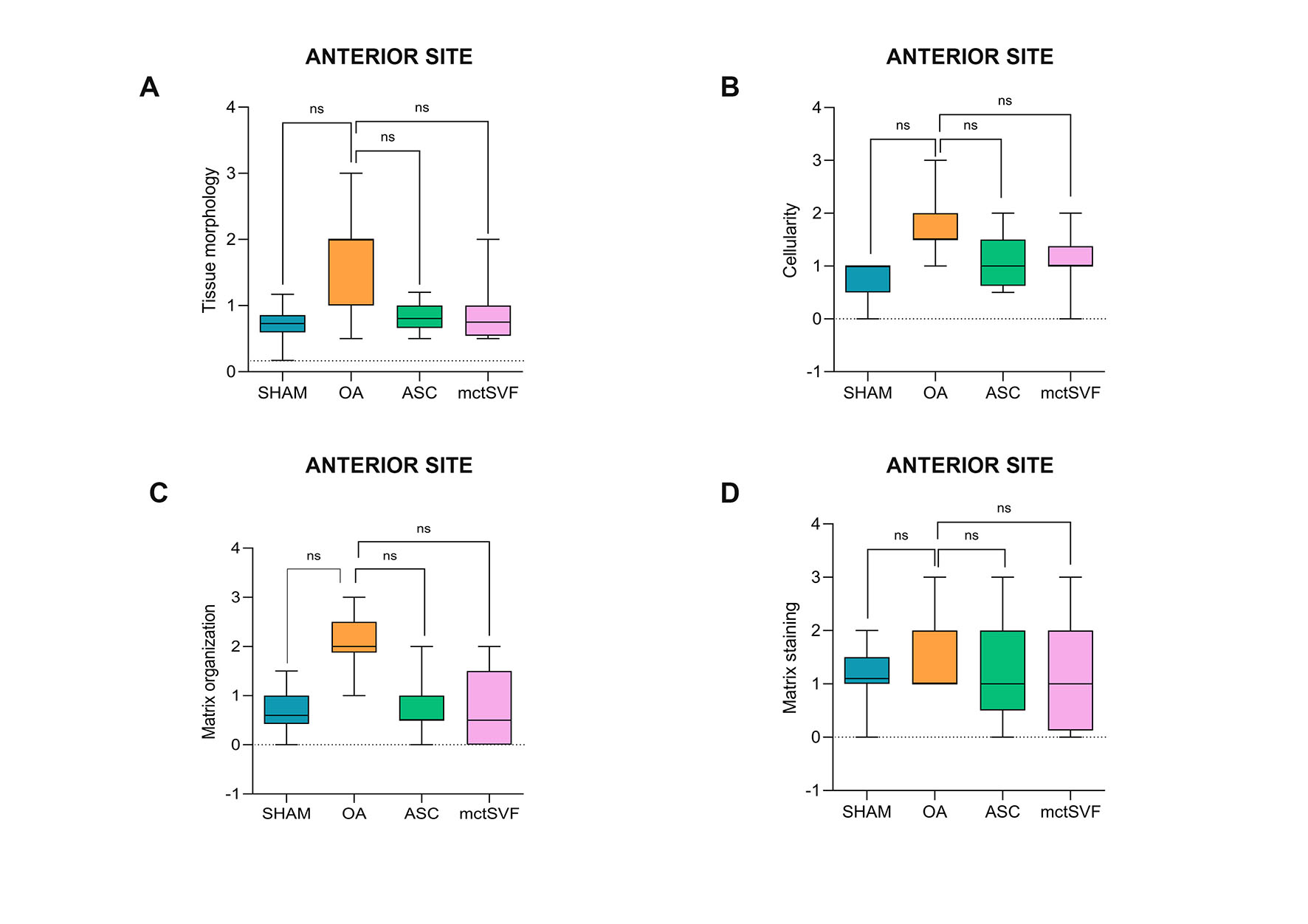

Supplement: Supplementary file 5 [file Image4.jpeg]

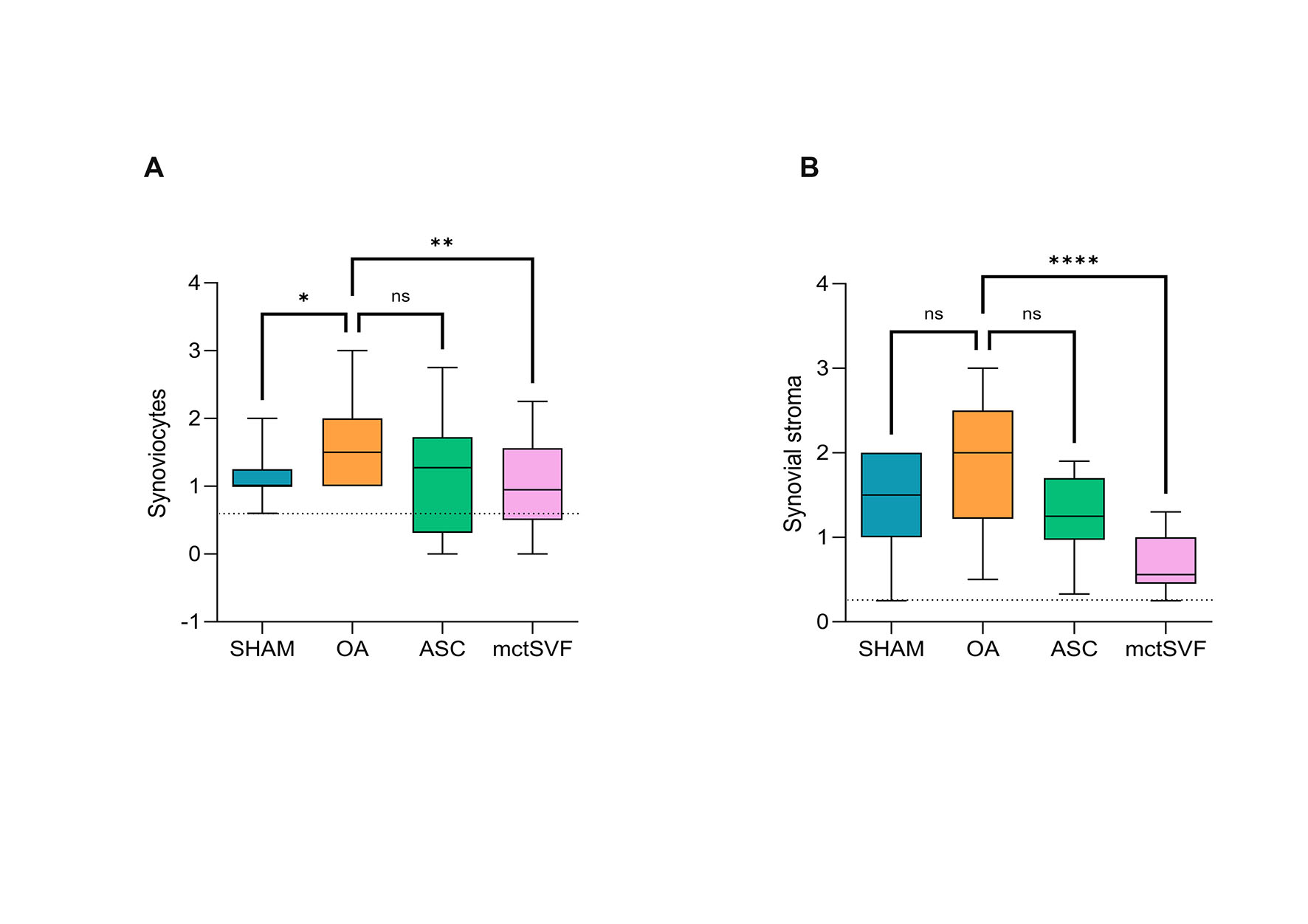

Supplement: Supplementary file 6 [file Image2.jpeg]

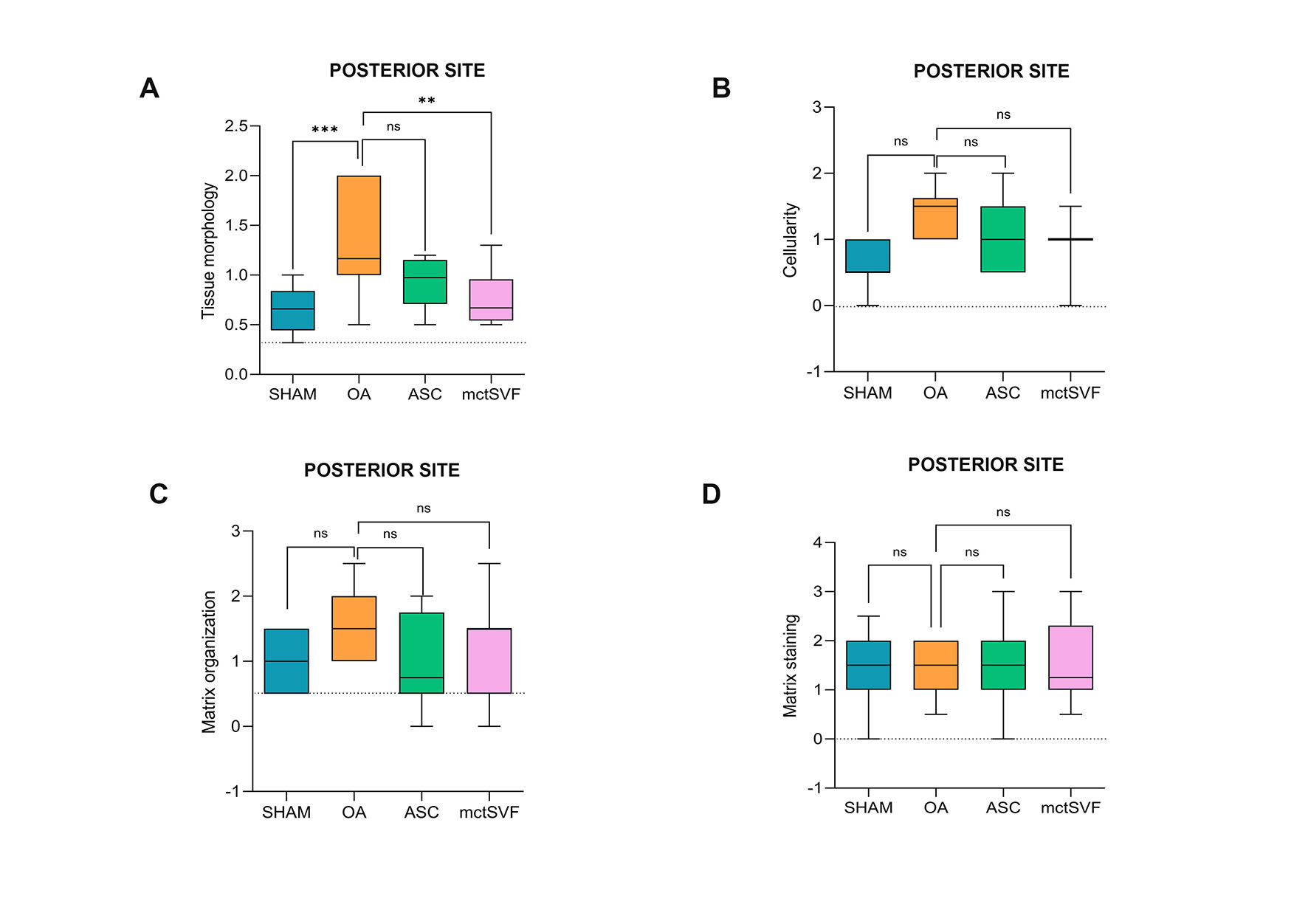

Supplement: Supplementary file 7 [file Image5.jpeg]
