# Supplementary material for: 3D connective micro-fragment enriched with stromal vascular fraction in osteoarthritis: chondroprotective evidence in a preclinical in vivo model
Source: Front Cell Dev Biol. 2025 Feb 27;13:1533405. doi: 10.3389/fcell.2025.1533405 (PMC11903414; doi:10.3389/fcell.2025.1533405)
Supplement: Supplementary file 3 [file DataSheet4.pdf]

Group	(I) factor 1	(J) factor 2	Average difference (I-J)	Sign.[b]	95% Confidence Interval[b]	
					Lower limit	Upper limit
**ASC**	cartilage	synovium	**29,295**	**,000**	18,656	39,935
		meniscus	**28,309**	**,000**	21,477	35,142
		ligament	**14,289**	**,018**	2,506	26,072
		synovial fluid	**27,686**	**,018**	4,820	50,552
	synovium	meniscus	-,986	1,000	-7,251	5,280
		ligament	**-15,006**	**,007**	-25,461	-4,552
		synovial fluid	-1,609	1,000	-19,200	15,982
	meniscus	ligament	**-14,020**	**,010**	-24,381	-3,660
		synovial fluid	-,624	1,000	-18,460	17,213
	ligament	synovial fluid	13,397	,399	-9,279	36,073
**mctSVF**	cartilage	synovium	**33,964**	**,000**	25,941	41,988
		meniscus	**25,836**	**,000**	19,774	31,898
		ligament	**22,994**	**,001**	10,837	35,151
		synovial fluid	**26,708**	**,012**	6,128	47,288
	synovium	meniscus	**-8,129**	**,003**	-13,166	-3,091
		ligament	**-10,970**	**,092**	-23,442	1,502
		synovial fluid	-7,257	,635	-22,396	7,883
	meniscus	ligament	-2,841	,988	-14,420	8,737
		synovial fluid	,872	1,000	-15,163	16,908
	ligament	synovial fluid	3,714	1,000	-20,469	27,897
		Synovial fluid	**-9,464**	**,033**	-18,191	-,737
Based on estimated marginal averages						
b. Adaptation for multiple comparisons: Sidak.						

Supplementary Table 4. Pairwise analysis with Sidak's test for multiple comparisons for biodistribution analysis in the ASC and mctSVF groups in the various joint tissues analysed.
