# Supplementary material for: 3D connective micro-fragment enriched with stromal vascular fraction in osteoarthritis: chondroprotective evidence in a preclinical in vivo model
Source: Front Cell Dev Biol. 2025 Feb 27;13:1533405. doi: 10.3389/fcell.2025.1533405 (PMC11903414; doi:10.3389/fcell.2025.1533405)
Supplement: Supplementary file 8 [file DataSheet3.pdf]

		Media	Standard Deviation	Sign. between groups[b]	95% confidence interval		Min	Max
					Lower limit	Upper limit		
% Biod. Cartilage	ASC	39,92	6,13	0,048	34,79	45,04	30,72	49,90
	mctSVF	41,90	5,09		37,64	46,16	35,48	49,95
% Biod. Sinovium	ASC	10,620	3,56	0,392	7,65	13,60	5,55	15,22
	mctSVF	7,94	2,50		5,85	10,03	4,37	11,32
% Biod. Meniscus	ASC	11,61	2,59	0,024	9,44	13,77	6,60	14,17
	mctSVF	16,06	2,03		14,36	17,77	13,05	19,02
% Biod. Ligament	ASC	25,63	6,51	0,001	20,19	31,07	18,71	34,88
	mctSVF	18,91	7,79		12,39	25,42	7,63	30,81
% Biod. Synovial fluid	ASC	12,23	11,02	0,278	3,02	21,44	,000	32,33
	mctSVF	15,19	10,38		6,52	23,87	3,91	33,67

Supplementary Table 3. Descriptive analysis of % cell biodistribution in the ASC and mctSVF in cartilage, synovium, meniscus, ligament and synovial fluid reported as mean, standard deviation and 95% confidence intervals (lower and upper limit).
